# Using Wearable Sensors for Sex Classification and Age Estimation from Walking Patterns

**DOI:** 10.3390/s25113509

**Published:** 2025-06-02

**Authors:** Rizvan Jawad Ruhan, Tahsin Wahid, Ashikur Rahman, Abderrahmane Leshob, Raqeebir Rab

**Affiliations:** 1 Department of Computer Science and Engineering, Bangladesh University of Engineering and Technology, Dhaka 1000, Bangladesh; rizvanjawad@gmail.com (R.J.R.); tahsinwahid2002@gmail.com (T.W.); 2 Department of Analytics, Operations, and Information Technology, University of Quebec in Montreal, Montreal, QC H2X 3X2, Canada; leshob.abderrahmane@uqam.ca; 3 Department of Computer Science and Engineering, Ahsanullah University of Science & Technology, Dhaka 1208, Bangladesh; raqeebir.cse@aust.edu

**Keywords:** gait analysis, smartphone, wearable sensor, motion tracking, machine learning, sex classification, age estimation

## Abstract

Gait refers to the walking pattern of an individual and it varies from person to person. Consequently, it can be considered to be a biometric feature, similar to the face, iris, or fingerprints, and can easily be used for human identification purposes. Person identification using gait analysis has direct applications in user authentication, visual surveillance and monitoring, and access control—to name a few. Naturally, gait analysis has attracted many researchers both from academia and industry over the past few decades. Within a small population, the accuracy of person identification could be very high; however, with the growing number of people in a given gait database, identifying a person only from gait becomes a daunting task. Hence, the focus of researchers in this field has exhibited a paradigm shift to a broader problem of sex and age prediction using different biometric parameters—with gait analysis obviously being one of them. Recent works on sex and age prediction using gait pattern obtained from the inertial sensors lacks an analysis of the features being used. In this paper, we propose a number of features inherent to gait data and analyze key features from the time–series data of accelerometer and gyroscopes for the automatic recognition of sex and the prediction of age. We have trained various traditional machine learning models and achieved the highest accuracy of 94% in sex prediction and an $R^{2}$ score of 0.83 in age estimation.

## 1. Introduction

**Figure 1:**
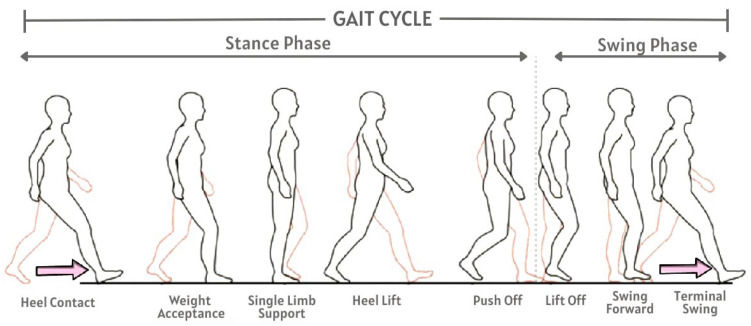
Gait cycle dynamics.

Gait is defined as cyclical locomotion achieved through walking that includes movements of arms, legs, hips, feet, and other upper and lower limbs [1]. The periodic movement of the legs is fundamental to the cyclic nature of human gait. Each gait period can be divided into two phases and eight configurations [2], as illustrated in Figure 1. In fact, there are 24 different components to human gait that can uniquely identify a particular individual [3]. Thus, when faced with a task of identifying a person out of *N* people, even a simple gait analysis might provide unique pattern for accurate enough result with small values of *N*. However, if the gait database grows with the inclusion of many individuals, identifying a person only from gait analysis might become a challenging task. In those situations, gait can only be used to narrow down the search space by filtering individuals based on some other factors, such as sex or age group, and generate a smaller set of potential candidates for further exploration.

Current state-of-the-art techniques for gait analysis can be broadly classified into four categories: vision-based, environment-based, RF-based, and wearable-sensor-based [4]. These solutions, except the wearable-sensor-based one, require a controlled research facility and expensive equipment (cameras, floor-deployed sensors, etc.) for their analysis, limiting their applicability in external/outdoor environments. On the other hand, wearable sensors are cheap and readily available in various types of devices, such as smartphones and smartwatches. So, these sensors can be easily used outside controlled environments while the user is performing daily activities naturally.

Gait analysis has many applications in security [5,6,7,8], healthcare [9,10,11], and fitness and sports [12,13,14]. In particular, estimation of an individual’s age and sex using wearable sensors can be useful in a wide variety of applications such as smart environments, healthcare, and soft biometric authentication systems [15]. For example, in a smart space, the functionality of AI-driven devices coupled with wearable sensors for age and sex detection can be adjusted based on a person’s sex or age group, providing better user experience. User recognition performance can be improved by combining traditional biometric traits with soft biometric attributes which includes sex and age. Additionally, law enforcement agencies can also use sensors of smartphones or other wearable devices to identify the sex or age of suspected individuals to narrow down the search space. In access control system installed in places like shopping malls, airports, liquor shops, and public clubs can utilize wearable sensors to detect age group and act accordingly.

Each person has unique characteristics in their walking pattern, but certain traits are consistently observed across demographic factors such as sex and age. For instance, adult males typically exhibit longer strides and greater step intervals compared to females, who tend to have shorter, quicker steps. These differences can be quantified by analyzing peak intervals in net acceleration signals. Similarly, rotational motion patterns also differ; adult females often display more frequent lateral twisting during walking, which can be captured via gyroscopic measurements. Gait patterns also evolve with age: children show short and inconsistent strides, adults develop stable and consistent patterns, and older individuals often demonstrate reduced step strength and less upper-body swing due to age-related physical decline. These biomechanical distinctions form the basis for many features used in sex and age prediction.

Several studies have explored sex and age prediction using wearable sensor data. Jain et al. [16] collected gait data from 109 subjects using two different smartphones under three walking conditions: slow, fast, and normal speeds. They extracted features using the Histogram of Oriented Gradients (HOG) method. Their method achieved a peak accuracy of 94.44% for fast walking and 91.07% for normal walking speed with bootstrap aggregating classifier. Sabir et al. [17] developed a gait database consisting of 30 male and 30 female participants walking while holding a smartphone. They extracted a total of 38 features and achieved the highest accuracy of 94.11% with an RNN-LSTM model.

Davarci et al. [18] collected data from 100 participants engaged in various activities and extracted feature sets by analyzing correlations between accelerometer data and users’ tap behavior to identify sex-discriminative patterns. Their proposed hybrid model achieved an 85% accuracy for sitting and standing activities and 83% for walking. Meena et al. [19] used a subset of 109 subjects from the OU-ISIR inertial gait dataset [20], where the data are manually extracted from a Motorola smartphone’s inbuilt accelerometer sensor. They reduced triaxial acceleration data into one dimension using PCA [21] and generated unique gait cycles for every individual. Their results showed that bagging trees achieved the highest accuracy at 96.3%.

Khabir et al. [22] used the OU-ISIR inertial gait dataset. They segmented each sample with a 3 s sliding window with 50% overlap and extracted a total of 88 features. Their approach achieved an accuracy of 84.76% with SVM classifier for sex classification and a $R^{2}$ score of 0.64 with a decision tree regressor for age estimation. Pathan et al. [23] also used a subset of OU-ISIR inertial gait dataset consisting of total 1556 samples. They extracted 84 features after applying NCA [24] and achieved an accuracy of 87.85% with bagging trees for sex prediction.

Ahad et al. [25] organized the GAG 2019 challenge using a large-scale OU-ISIR inertial dataset that included 745 participants ranging from 2 to 78 years old. They evaluated both traditional handcrafted features and modern deep learning approaches, such as angle-embedded gait dynamic images and temporal convolutional networks (TCNs). Their best-performing deep model achieved a mean absolute error (MAE) of 5.39 years for age estimation and a 24.23% error rate for sex prediction, highlighting the potential of deep models. Van Hamme et al. [26], winners of the GAG 2019 challenge, showed that even short gait segments can yield accurate predictions when using well-preprocessed data. They compared handcrafted and deep learning methods, highlighting the effectiveness of sensor-invariant features and temporal convolutional networks.

Existing studies [16,17,18,19,22,23,25,26] typically emphasize predictive accuracy and employ ‘black-box’ models, rather than interpreting which specific gait features drive the predictions. Moreover, many of these works rely on relatively simple statistical features or dimensionality-reduced data, which limit both interpretability and the richness of gait representation. Thus, there is a clear gap in the literature: no recent study has provided a detailed and systematic analysis of which gait characteristics are most influential in predicting sex and age from wearable sensors. We address this gap by identifying and extracting a diverse and informative set of handcrafted features from the time–series signals of triaxial acceleration and rotational velocity, captured by accelerometer and gyroscope sensors. Our feature set spans time, frequency, jerk, and correlation domains, going beyond conventional measures to capture subtle and higher-order variations in gait. This allows for a deeper understanding of the relationship between motion dynamics and demographic attributes.

Our work aims to provide a simple yet powerful methodology for sex and age recognition using wearable sensors, combining improved prediction accuracy with enhanced interpretability. We analyze and explain the significance of the extracted features in the context of human biomechanics. In addition, we evaluate the performance of several traditional machine learning models using this feature set and conduct comparative analyses across different age groups and sensor placements. The experimental results show that our proposed system achieves a highest accuracy of 94% in sex prediction and an $R^{2}$ score of 0.83 in age estimation, outperforming existing research works in this domain.

The road map of the paper is as follows. Section 1 already includes the research works that are related to this work. Section 2 provides data collection methodology, working principal, extracted features and their relevance. Section 3 provides the experimental results, result analyses, and performance comparison. Finally, Section 6 concludes the paper with some pointers to future works.

## 2. Methods and Data

The goal of our research is to accurately estimate sex and age from walking patterns captured using wearable sensors. By analyzing key features in the data, we aim to uncover patterns that vary with age and sex. Sensors like accelerometers and gyroscopes can be placed on various parts of the body—such as the foot, ankle, knee, or waist—to record motion data. In our study, we focused on signals captured from the waist. The raw sensor data were first filtered and then segmented using a sliding window approach. We extracted meaningful features from time, frequency, jerk, and correlation domains, and applied Recursive Feature Elimination (RFE) to select the top 30 most relevant features for each task. Then, a range of machine learning models was then trained using 5-fold cross-validation. We report model performance in terms of classification accuracy for sex prediction and $R^{2}$ score for age estimation.

### 2.1. Methodology

**Figure 2:**
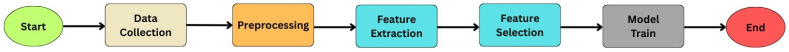
Training the data for our model.

**Figure 3:**
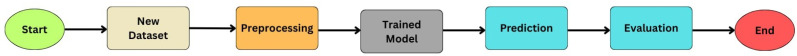
Testing and evaluating our model on new data.

First, we collect raw time–series data using accelerometer and gyroscope. Then, we preprocess the raw data and remove any noise using a filtering method. Then, we extract the features and apply a feature selection technique to select the best features. Finally, we construct the training and test set using the selected feature set and train our models with the training set and evaluate on the test set. The following sections provide a detailed description of our method. The graphical summary of the proposed methodology is shown in Figure 2 and Figure 3.

### 2.2. Data Collection

**Figure 4:**
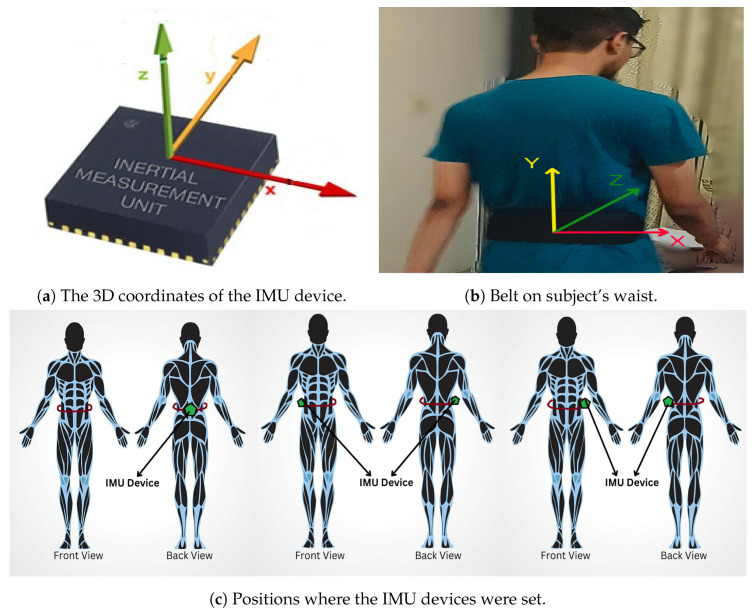
Setup of the gait-capturing system.

We can place the sensors on different parts of the human body such as foot, ankle, knee, and waist to identify gait pattern. For this paper, we used the OU-ISIR inertial gait sensor dataset [20], the largest inertial-sensor-based gait database, containing data from 744 subjects (389 males and 355 females) aged 2 to 78 years. Each subject walked a designed path with a belt around their waist with three IMU sensors and an Android device strapped on the belt, as shown in Figure 4. All the sensors collected samples at a frequency of 100 Hz.

We have used the samples of the partitions IMUZ center, right, and left, with level walking activity labels from both manual and automatic subsets of the dataset. We have excluded the data collected using Android, as they lack gyroscope readings.

### 2.3. Equipment

**Figure 5:**
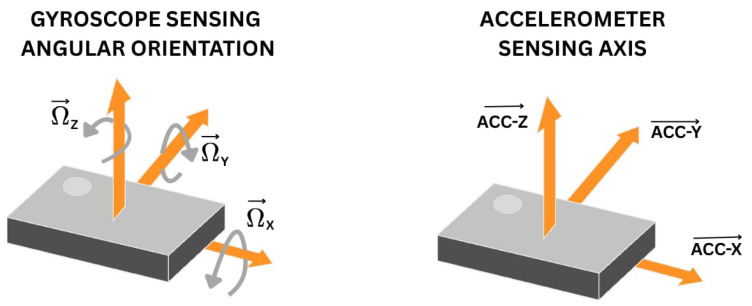
Gyroscope and accelerometer.

Gyroscopes and accelerometers are both MEMS devices which measure acceleration and rotational velocity, respectively, along the triaxial direction, as shown in Figure 5. A gyroscope is a sensor that measures rotational motion and orientation. A triaxial gyroscope detects rotation in three directions (X, Y, and Z), providing a complete view of movement. An accelerometer is a device that works by detecting changes in motion and converting them into electrical signals. These sensors are found in many devices which we use on a daily basis. For example, they are incorporated into smartphones, where the sensors help detect movement. These sensors are used in smartphones for screen rotation, gaming controllers for motion tracking, and vehicles and drones for stability.

To capture and analyze these variations in gait, we can use sensors like accelerometer and gyroscope and record triaxial acceleration and rotational speed of different parts of body while walking.

### 2.4. Data Preprocessing

**Figure 6:**
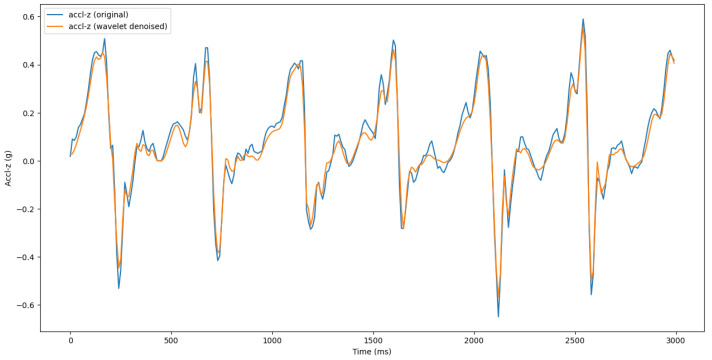
The effect of wavelet denoising of accl-z signal.

We have ensured that all the sensors’ readings are aligned with a common reference axis. We have used a sliding window of 3 s [27] with a 50% overlap to capture multiple gait cycles, reduce sensitivity to noise, and improve feature extraction. Since raw sensor readings often contain noise due to environmental factors, we have applied a signal processing technique called wavelet denoising [28] to enhance the quality of the signals. Wavelet denoising reduces noise by decomposing a signal into different frequency components using wavelet transform. It then applies a thresholding technique to selectively remove low-energy coefficients, which typically represent noise, while preserving high-energy coefficients that contain meaningful signal information. Finally, the signal is reconstructed, resulting in a cleaner version with reduced noise while retaining essential features. This method is particularly effective in preserving important features of the data while removing unwanted noise. Figure 6 shows an example of wavelet denoising.

### 2.5. Feature Extraction

Each sample in our dataset contains 6D components which are the triaxial values of acceleration (g) from accelerometer and rotational speed (rad/s) from gyroscope. We have added an additional component which is the net acceleration by calculating the vector sum of acceleration along the directions of the x, y, and z axes (lateral, vertical, and forward direction). We have extracted four categories of features: time domain, jerk domain (the rate of change with regard to time), frequency domain, and correlation features for each of the components. The time domain features are statistical values calculated directly from time–series data. The time domain features that we have extracted include mean, standard deviation, minimum, maximum, skewness, kurtosis, inter-quartile range, mean absolute deviation, peak per second, energy per second, average and std of peak value, and average and std of interval between peaks. The jerk domain is defined as the derivative or the rate of change of acceleration and rotational speed with respect to time. The jerk domain’s features are derived similarly to the time domain’s features, but are computed from the time derivatives of the original signals, capturing variations in motion more effectively. The frequency domain features are of two types: Fast Fourier Transform (FFT) and power spectral density (PSD). For FFT type, we convert time–series data to the frequency domain using the Fast Fourier Transform [29] and compute various features such as lower and upper bound of bandwidth, peak frequency mean, max, min, skewness, kurtosis, and spectral entropy of frequency. For PSD type, we estimate power spectral density (PSD) using Welch’s method [30] and extract similar statistical features of frequency.

As human walking frequency is usually limited within 20 Hz, we have defined six bands of frequency: Delta (0–3 Hz), Theta (3–6 Hz), Alpha (6–9 Hz), Beta (9–12 Hz), Gamma (12–15 Hz), and Zeta (15–18 Hz). We computed features like total power, percentage of total power, and max frequency for each band.

Lastly, correlation features include Spearman correlations [31] of every pair of components of a sample. Spearman correlation measures the monotonic relationship between two variables. Unlike Pearson correlation [32], it captures non-linear relationships, making it useful for analyzing data that do not follow a strict linear trend. By comparing the rankings of two variables rather than their actual values, the Spearman correlation coefficient (ρ) calculates the direction and strength of a monotonic relationship. It is calculated by converting raw numbers into ranks, calculating rank differences, and then using the differences to derive ρ.

Spearman correlation coefficient (ρ) is computed as:

$$\rho =1-\frac{6\sum d_{i}^{2}}{n(n^{2}-1)}$$

where

- $d_{i}$: difference between the ranks of the corresponding values in the two variables.
- *n*: number of observations.

A significant positive correlation is indicated by a (ρ) value around +1, whereas a strong negative correlation is indicated by a value near −1.

We have used abbreviated versions of each feature using the following notation: accl/gyro-x/y/z-feature_name, where accl refers to accelerometer and gyro refers to gyroscope. For example, gyro-y-bandwidth-psd-lower-bound refers to the lower bound of bandwidth in the psd domain of the gyroscope’s y-axis values. For correlation features, the notation accl/gyro-x/y/z-accl/gyro-x/y/z-spearman refers to the correlation between the accelerometer/gyroscope’s x/y/z-axis and the accelerometer/gyroscope’s x/y/z-axis values using the Spearman correlation coefficient. For example, the notation accl-x-gyro-x-spearman represents the Spearman correlation between the accelerometer’s x-axis and the gyroscope’s x-axis.

By extracting features separately from each axis (x, y, z) of both the accelerometer and gyroscope, we ensure that the model captures directional nuances of human motion. Each axis represents a different physical direction—lateral (x), vertical (y), and forward (z)—and exhibits unique patterns during walking. For instance, vertical acceleration reveals step force and lift, while forward acceleration captures stride tempo. Similarly, rotational patterns differ significantly between axes and provide insight into body twist or sway, which vary with sex and age. Including all components allows us to retain the full spatiotemporal richness of gait and improves both interpretability and model performance.

### 2.6. Feature Selection

Recursive Feature Elimination (RFE) recursively removes the least important features based on the weights assigned to it by the estimator model at each step and then re-ranks the remaining ones by retraining the estimator model based on the updated feature set [33]. We have applied RFE with a Logistic Regression Model to reduce the large feature set to only 30 features for sex prediction. Similarly, we have applied RFE with SVR to reduce the feature set to only 30 features for age estimation. Table 1 illustrates both feature sets. To provide clearer insights, we have shown analytical feature names to showcase what each feature captures, in Table 2 and Table 3. In the tables, we used the terms Forward Tilt, Lateral Twist, and Vertical Swing to denote the rotational motions around the x, y, and z axes, respectively.

### 2.7. Features Description

In this section, we describe how key features were engineered from the raw sensor data to support sex classification and age estimation. These features span multiple domains, including time domain, frequency domain, and jerk domain representations.

#### 2.7.1. Sex Classification

**Figure 7:**
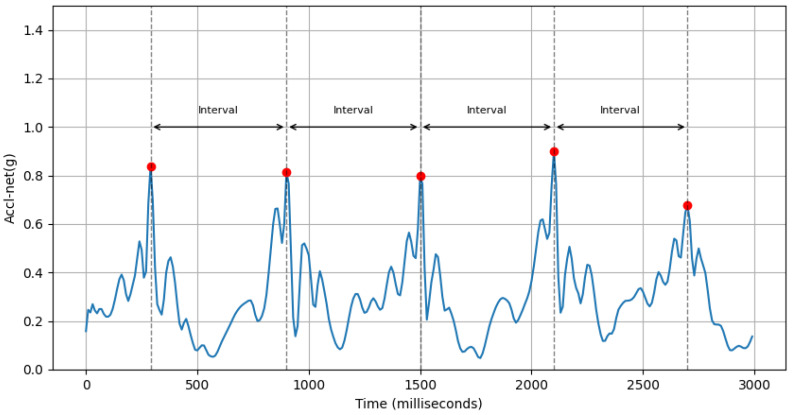
Positive peaks (red dots) of accl-net (blue line) and intervals between them.

**Figure 8:**
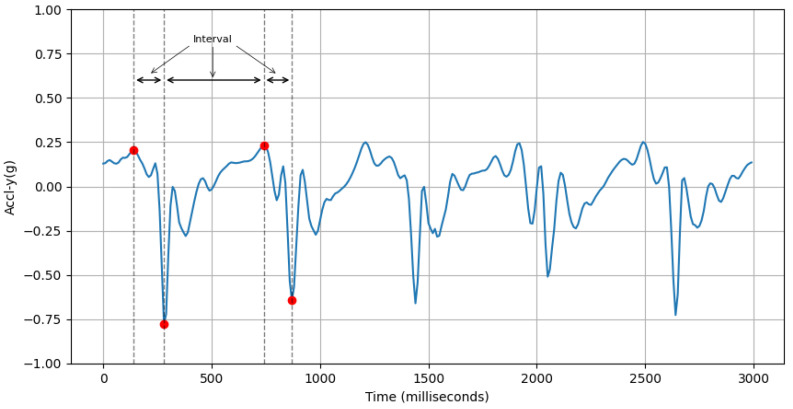
First few peaks (red dots) of accl-y (blue line) and the intervals between them.

**Figure 9:**
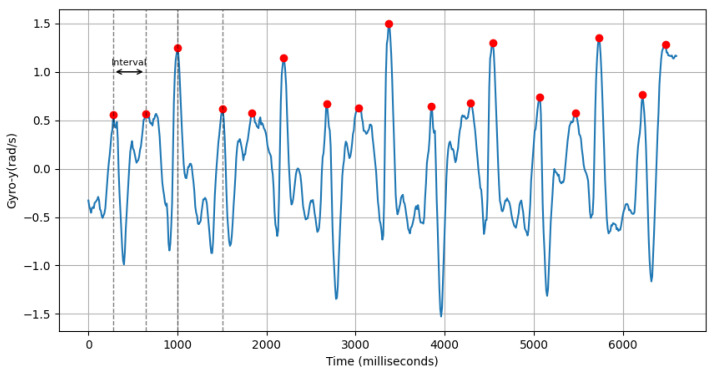
Positive peaks (red dots) of gyro-y (blue line) and interval between first two peaks.

**Figure 10:**
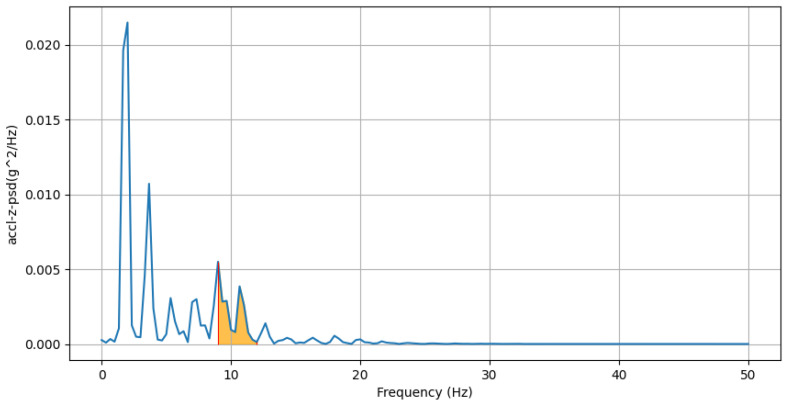
PSD domain of accl-z: area under beta band (9–12 Hz) is highlighted by orange, with red lines indicating the boundaries of the region. The blue line represents the PSD domain of accl-z across frequencies.

**Figure 11:**
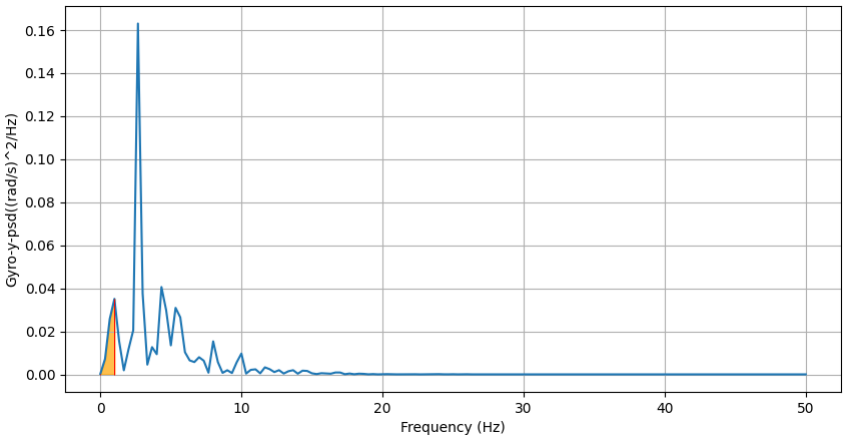
Lower bound of bandwidth of PSD domain of gyro-y: the blue line represents the PSD of gyro-y across frequencies. The red line marks the frequency corresponding to the lower bound, and the orange area highlights the area under the lower frequency band.

(a) **Avg Step Interval:** This feature calculates the average interval period (in milliseconds) between positive peaks of accl-net. An example of this is shown in Figure 7.
  $$\mathrm{accl}\text{-}\mathrm{net}\text{-}\mathrm{positive}\text{-}\mathrm{peak}\text{-}\mathrm{interval}\text{-}\mathrm{mean}=\frac{\sum_{i=2}^{n}\left(t_{i}-t_{i-1}\right)}{n}$$
  where $t_{i}$ is the time of the *i*th positive peak of accl-net, and *n* is the number of positive peaks.
(b) **Variability in Interval Between Peaks of Vertical Acceleration:** This feature computes the standard deviation of the interval period between peaks of accelerometer-y-axis values within a sample. An example of this is shown in Figure 8.
  $$\mathrm{accl}\text{-}y\text{-}\mathrm{peak}\text{-}\mathrm{interval}\text{-}\mathrm{std}=\sqrt{\frac{\sum_{i=2}^{n}{\left(t_{i}-t_{i-1}-\mathrm{accl}\text{-}y\text{-}\mathrm{peak}\text{-}\mathrm{interval}\text{-}\mathrm{mean}\right)}^{2}}{n}}$$
  where $t_{i}$ is the time of the *i*th peak of accl-y, *n* is the number of peaks, and accl-y-peak-interval-mean is the average interval period between peaks of accelerometer-y-axis values.
(c) **Avg Interval Between Peaks of Lateral Twist Motion:** This feature computes the average interval period between positive peaks of gyroscope-y-axis values within a sample. An example of this is shown in Figure 9.
  $$\mathrm{gyro}\text{-}y\text{-}\mathrm{positive}\text{-}\mathrm{peak}\text{-}\mathrm{interval}\text{-}\mathrm{mean}=\frac{\sum_{i=1}^{n}\left(t_{i}-t_{i-1}\right)}{n}$$
  where $t_{i}$ is the time of the *i*th positive peak of gyro-y, and *n* is the number of positive peaks.
(d) **Power Concentration of Forward Acceleration in Beta Band (9–12 Hz):** This feature computes the percentage of total power within the beta band (9–12 Hz) of the power spectral density (PSD) domain for accelerometer-z-axis values. An example of beta band in PSD domain of accl-z is shown in Figure 10.
  $$\mathrm{accl}\text{-}z\text{-}\mathrm{percentage}\text{-}\mathrm{power}\text{-}\mathrm{beta}\text{-}\mathrm{band}\text{-}\mathrm{psd}=\frac{\sum_{f=9}^{12}P_{z}\left(f\right)}{\sum_{f}P_{z}\left(f\right)}$$
  where $P_{z}\left(f\right)$ is the PSD value of the accl-z at frequency *f*.
(e) **Lower Bound of Bandwidth of Lateral Twisting Motion:** This feature computes the frequency value where the cumulative power exceeds 10% of the total power in the PSD domain for the gyroscope-y-axis. You can find an example of this in Figure 11.
  $$f_{\mathrm{lower}}=\min\left\{f:\frac{\sum_{0}^{f}P_{y}\left(f\right)}{\sum_{f}P_{y}\left(f\right)}\geq 0.1\right\}$$
  where $P_{y}\left(f\right)$ is the PSD value of gyro-y at frequency *f*.

#### 2.7.2. Age Estimation

**Figure 12:**
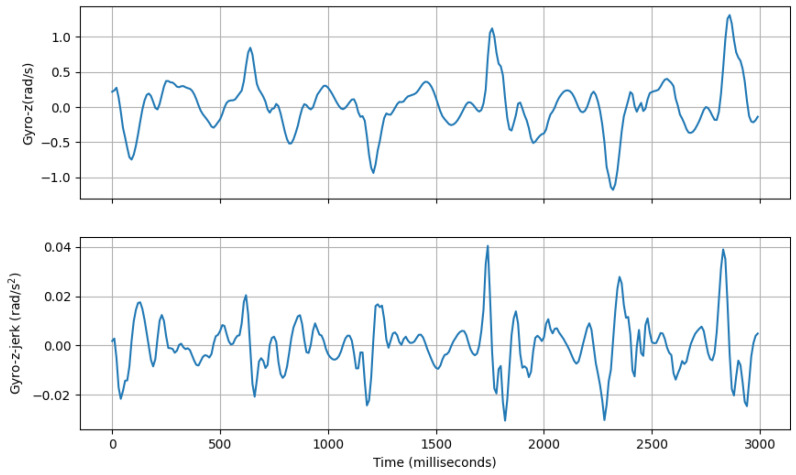
An example of Gyro-z signal (**top**, blue line) and its jerk (**bottom**, blue line).

**Figure 13:**
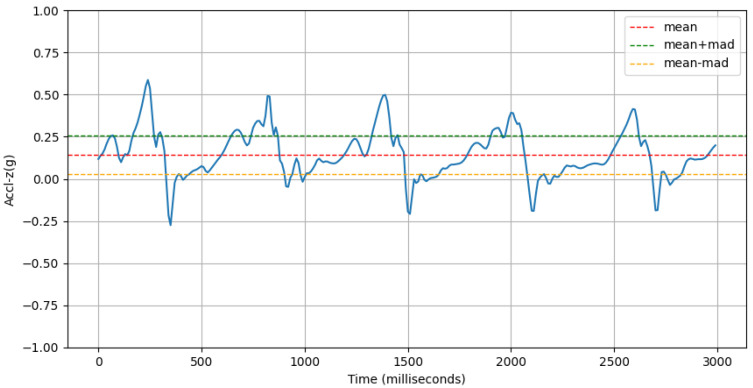
An example of accl-z signal (blue line) and its MAD.

**Figure 14:**
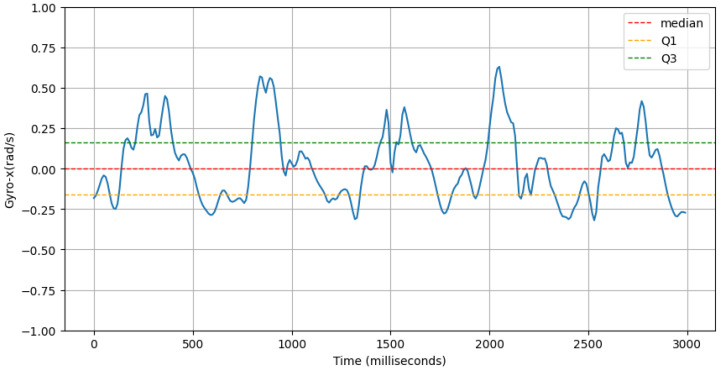
An example of Gyro-x signal (blue line) along with its first quartile (Q1, orange dashed line) and third quartile (Q3, green dashed line).

**Figure 15:**
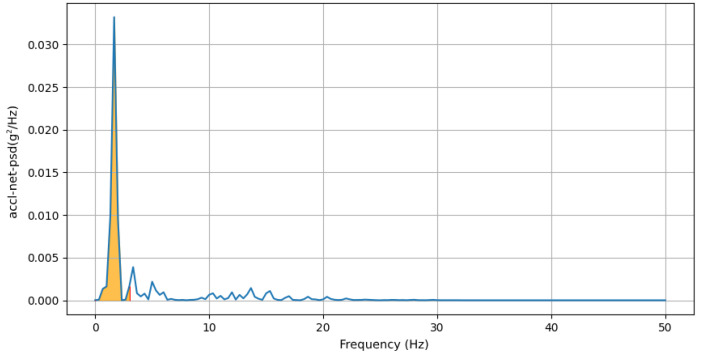
PSD domain of accl-net: area under delta band (0–3 Hz) is highlighted by orange, with red lines indicating the boundaries of the delta band region. The blue line represents the PSD domain of accl-z across frequencies.

(a) **Avg Step Interval:** This feature calculates the average interval period (in milliseconds) between positive peaks of accl-net as described previously.
(b) **Range of rate of change of Vertical Swing Motion:** This feature computes the inter-quartile range of the derivative of gyroscope-z signal with regard to time. An example of jerk of gyro-z is shown in Figure 12.
  $$\mathrm{gyro}\text{-}z\text{-}\mathrm{jerk}\text{-}\mathrm{iqr}=Q_{3}\left(\mathrm{gyro}\text{-}z\text{-}\mathrm{jerk}\right)-Q_{1}\left(\mathrm{gyro}\text{-}z\text{-}\mathrm{jerk}\right)$$
  where gyro-z-jerk is the derivative of the gyroscope-z signal with regard to time, $Q_{3}$ is the third quartile (75th percentile), and $Q_{1}$ is the first quartile (25th percentile) of the jerk signal.
(c) **Deviation of Forward Acceleration:** This feature computes the mean absolute deviation (MAD) of the accelerometer-z signal. An example of this is shown in Figure 13.
  $$\mathrm{accl}\text{-}z\text{-}\mathrm{mad}=\frac{1}{n}\sum_{i=1}^{n}\left|{\mathrm{accl}\text{-}z}_{i}-\mu \right|$$
  where ${\mathrm{accl}\text{-}z}_{i}$ is the *i*-th value of the accl-z signal and μ is the the mean of the accl-z signal.
(d) **Range of Forward Tilt Motion:** This feature computes the inter-quartile range of the gyroscope-x signal. An example of this is shown in Figure 14.
  $$\mathrm{gyro}\text{-}x\text{-}\mathrm{iqr}=Q_{3}\left(\mathrm{gyro}\text{-}x\right)-Q_{1}\left(\mathrm{gyro}\text{-}x\right)$$
  where gyro−x represents the gyroscope-x signal, $Q_{3}$ is the third quartile (75th percentile), and $Q_{1}$ is the first quartile (25th percentile) of the gyroscope-x signal.
(e) **Power Concentration of Net Acceleration in Delta Band (0–3 Hz):** This feature computes the percentage of total power within the delta band (0–3 Hz) of the power spectral density (PSD) domain for accl-net signal. An example of delta band in PSD domain of accl-net is shown in Figure 15.
  $$\mathrm{accl}\text{-}\mathrm{net}\text{-}\mathrm{percentage}\text{-}\mathrm{power}\text{-}\mathrm{delta}\text{-}\mathrm{band}\text{-}\mathrm{psd}=\frac{\sum_{f=0}^{3}P_{\mathrm{net}}\left(f\right)}{\sum_{f}P_{\mathrm{net}}\left(f\right)}$$
  where $P_{z}\left(f\right)$ is the PSD value of the accl-net at frequency *f*.

## 3. Results

### 3.1. Features Analysis

In this section, we explore how the key features described in Section 2.7 help distinguish between different sexes and age groups. We interpret their significance by analyzing how these features vary across age and sex using the OU-ISIR inertial gait dataset.

#### 3.1.1. Sex Classification

**Figure 16:**
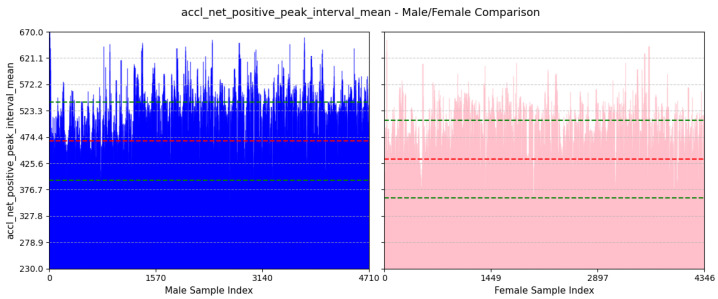
Comparison of accl-net-positive-peak-interval-mean between male and female participants. The red dashed line indicates the mean value for this feature, while the green dashed lines represent the mean ± standard deviation.

**Figure 17:**
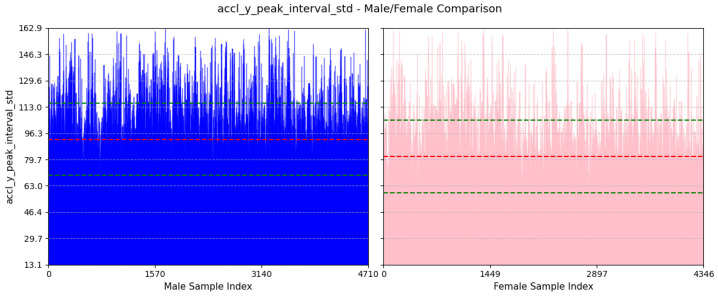
Comparison of accl-y-peak-interval-std between male and female participants. The red dashed line indicates the mean value for this feature, while the green dashed lines represent the mean ± standard deviation.

**Figure 18:**
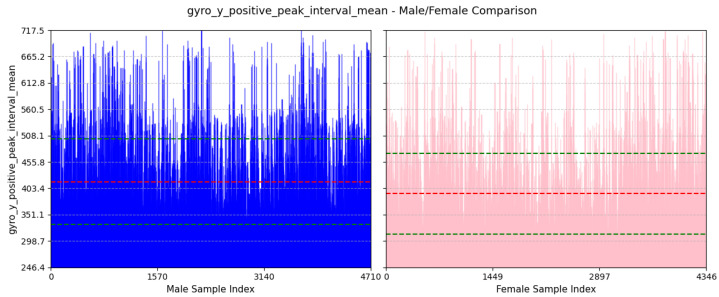
Comparison of gyro-y-positive-peak-interval-mean between male and female participants. The red dashed line indicates the mean value for this feature, while the green dashed lines represent the mean ± standard deviation.

**Figure 19:**
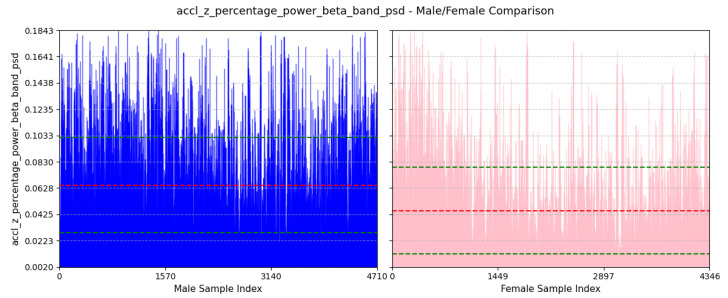
Comparison of accl-z-percentage-power-beta-band-psd between male and female participants. The red dashed line indicates the mean value for this feature, while the green dashed lines represent the mean ± standard deviation.

**Figure 20:**
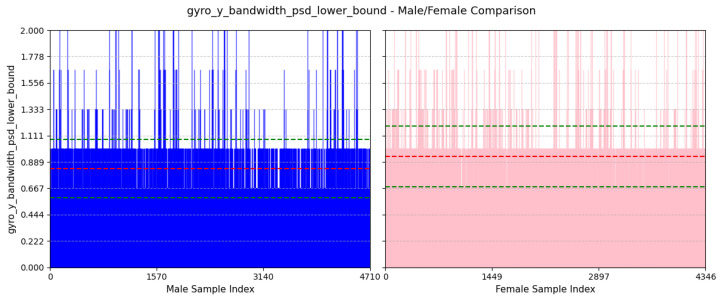
Comparison of gyro-y-bandwidth-psd-lower-bound between male and female participants. The red dashed line indicates the mean value for this feature, while the green dashed lines represent the mean ± standard deviation.

(a) **Avg Step Interval:** We can see from Figure 16 that the average value of this feature for males is 466.81±72.35ms and females is 433.44±72.1ms. This is because males generally have longer strides, leading to a greater interval between positive peaks. On the other hand, females usually take smaller steps.
(b) **Variability in Interval Between Peaks of Vertical Acceleration:** Based on Figure 17, the average value of this feature for males is 92.47±22.8ms and that for females is 81.71±22.94ms. This feature assesses the variability in the timing of peaks in acceleration along the y-axis. A higher standard deviation for males suggests a more dynamic movement in the y-axis, whereas a lower standard deviation for females implies a more stable movement in the y-axis.
(c) **Avg Interval Between Peaks of Lateral Twist Motion:** As depicted in Figure 18, the average value of this feature for males is 416.47±85.18ms and that females is 392.87±80.19ms. A positive peak in the gyroscope Y-axis may suggest a twisting motion of the torso to the right. Males typically have longer and wider strides, resulting in longer intervals between gyroscope Y-axis positive peaks. On the other hand, smaller strides in females may be the cause for shorter intervals between positive peaks of gyro-y.
(d) **Power Concentration of Forward Acceleration in Beta Band (9–12 Hz):** We can see from Figure 19 that the average value of this feature for males is 6.5%±3.66% and that for females is 4.54%±3.32%. Most of the power of human walking motion is within the delta band (0–3 Hz), so this feature highlights that higher frequency component is comparatively more dominant in males than females for acceleration along the z-axis. Males normally walk with faster and stronger pace, which creates a more dynamic movement, increasing the percentage of power within the beta band (9–12 Hz).
(e) **Lower Bound of Bandwidth of Lateral Twisting Motion:** We can see from Figure 20 that the average value of this feature for males is 0.83±0.25Hz and that for females is 0.94±0.26Hz. This feature assesses the initial frequency at which significant energy associated with twisting movement around the y-axis begins. A higher lower bound in females suggests a more frequent twisting motion around the y-axis. A longer and wider stride in males may contribute to a twisting motion of less frequency.

#### 3.1.2. Age Estimation

**Figure 21:**
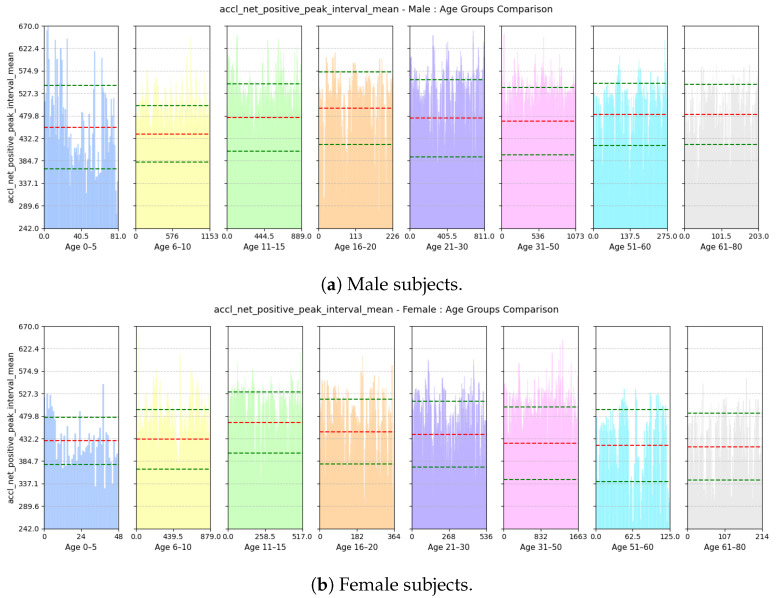
Comparison of accl-net-positive-peak-interval-mean between age groups for (**a**) male and (**b**) female subjects. The red dashed line indicates the mean value for this feature, while the green dashed lines represent the mean ± standard deviation.

**Figure 22:**
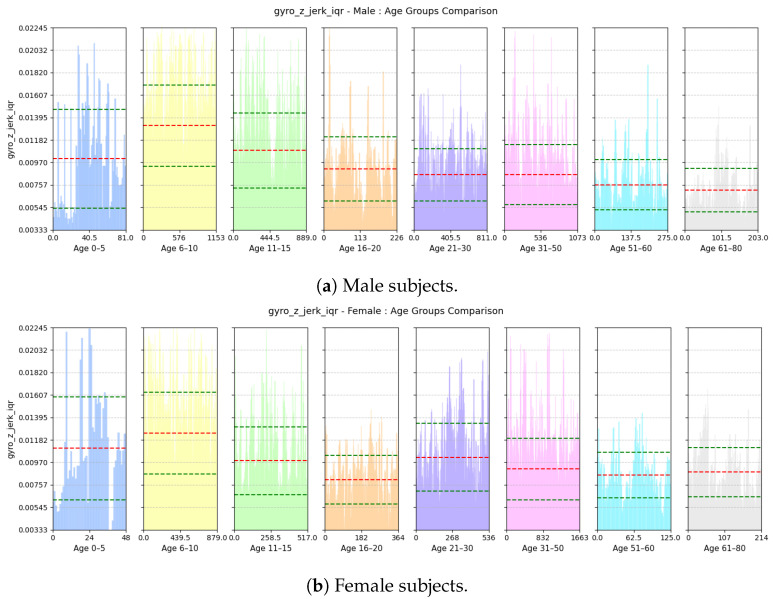
Comparison of gyro-z-jerk-iqr between age groups for (**a**) male and (**b**) female subjects. The red dashed line indicates the mean value for this feature, while the green dashed lines represent the mean ± standard deviation.

**Figure 23:**
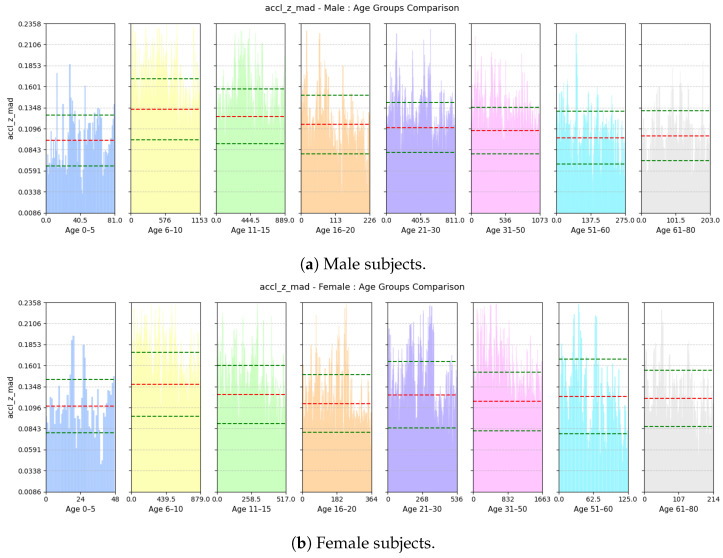
Comparison of accl-z-mad between age groups for (**a**) male and (**b**) female subjects. The red dashed line indicates the mean value for this feature, while the green dashed lines represent the mean ± standard deviation.

**Figure 24:**
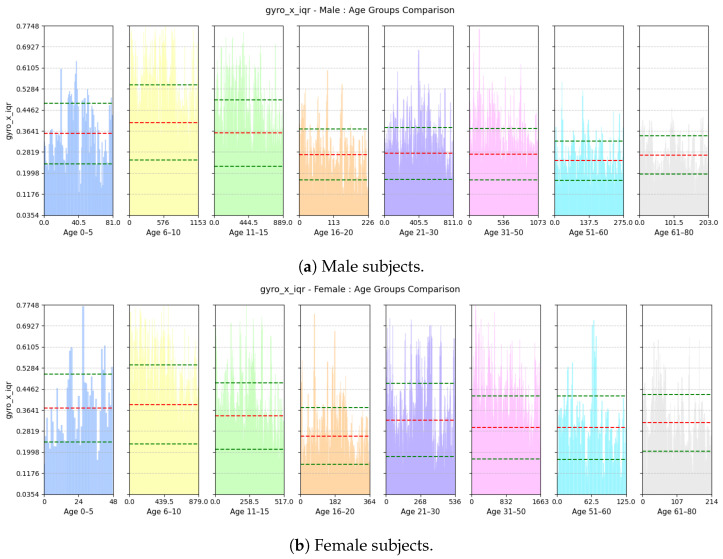
Comparison of gyro-x-iqr between age groups for (**a**) male and (**b**) female subjects. The red dashed line indicates the mean value for this feature, while the green dashed lines represent the mean ± standard deviation.

**Figure 25:**
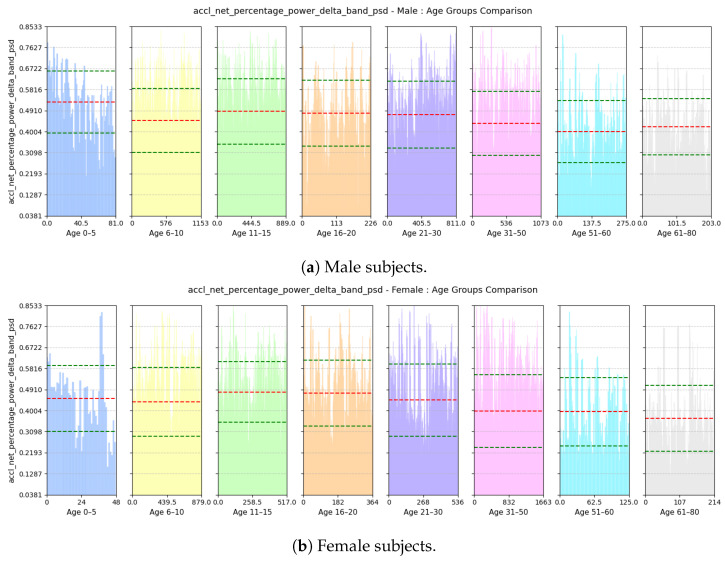
Comparison of accl-net-percentage-power-delta-band-psd between age groups for (**a**) male and (**b**) female subjects. The red dashed line indicates the mean value for this feature, while the green dashed lines represent the mean ± standard deviation.

(a) **Avg Step Interval:** Based on Figure 21, we see that both male and female children from ages 0 to 10 have lower interval period, as children tend to take small steps; the standard deviation of this feature is quite high among the male children. However, for males, the interval period gradually increases until the age of 20; this is because, as they grow older and taller, their strides also become longer. Then, it decreases slightly and stabilizes until the age of 50. Then, as they grow older and their walking pace gradually slows down, the interval between positive peaks also become longer from 51 to 60 years of age. For females, the interval period gradually increases until the age of 15 and then stabilizes until the age of 30; then, it keeps on decreasing up to the age of 80. Pregnancy and other reasons may cause biological changes to females, resulting in their strides becoming smaller.
(b) **Range of rate of change of Vertical Swing Motion:** This feature assesses the variability in the rate of change of rotational motion around the z-axis, i.e., swinging motion while walking. Based on Figure 22, we see that variability in the jerk is high for both males and females aged from 0 to 10 as children tend to have very unstable walking style and they have more swinging in their walking style. The iqr value decreases gradually with increases in age for both males and females as their walking style becomes more stable.
(c) **Deviation of Forward Acceleration:** Based on Figure 23, we see that the MAD of accl-z is highest in the group aged 6–10 years due to their volatile walking pace. The deviation decreases gradually with increasing age for both males and females as their walking pace becomes more stable.
(d) **Range of Forward Tilt Motion:** This feature assesses the variability in rotational speed around the x axis which basically emphasizes the importance of tilting motion in the forward and backward direction while walking. Based on Figure 24, we see that the tilting movement stays significant throughout the growing years, continuing until age 15 for both males and females. However, then it gradually goes down and remains stable until age of 80.
(e) **Power Concentration of Net Acceleration in Delta Band (0–3 Hz):** This feature assesses the percentage of power within delta band (0–3 Hz) which is the dominant band as the walking frequency of humans is around 1 Hz. Based on Figure 25, we see that the percentage of power is lower for the group aged 6–10 years due to their erratic gait pattern. As their walking pattern stabilizes, the percentage of power increases and remains steady from ages 11 to 30 for both males and females. However, it declines progressively from ages 31 to 80, which is attributed to slower walking paces and weaker strides with advancing age.

### 3.2. Model Training

**Figure 26:**
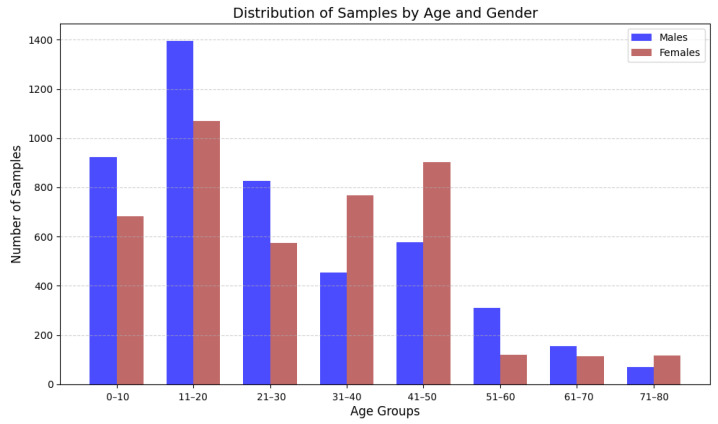
Age group and sex distribution.

We have split the dataset into two parts: 70% for the training set and 30% for the test set. After preprocessing and feature extraction, the training set contains 6339 samples and the test set contains 2717 samples. The dataset has an imbalance in age distribution, as shown in Figure 26.

For the training phase, we used 5-fold cross-validation for tuning the hyperparameters of our models. Finally, we evaluated our trained models on the unseen test set. In the following subsections, we describe the classifier and regressor models.

### 3.3. Classifier Models

For the classification of sex, we have trained a set of widely used classifiers: Logistic Regression (LR) [34], k-Nearest Neighbors (kNN) [35] classifier, Support Vector Classifier (SVC) [36], Random Forest (RF) [37], eXtreme Gradient Boosting (XGBoost) [38], Light Gradient Boosting Machine (LightGBM) [39], and a Stacking Ensemble [40].

The Stacking Ensemble uses SVC, kNN, and Random Forest as the base models, with LightGBM as the meta-model. The ensemble is implemented using ‘passthrough=True’, which means that both the original input feature vector and the base model outputs are used as input to the meta-model.

During training, each base model is trained on the original feature set using cross-validation folds. For each fold, the base models generate out-of-fold predictions for the validation data. These predictions are then concatenated with the original features of each sample to form an extended feature vector. This new set of vectors is used to train the meta-model, LightGBM, which learns how to optimally combine the base model outputs and the original features for final prediction.

In the test phase, an unseen sample is passed through the three trained base models to generate their predictions. These predictions are again concatenated with the original input feature vector of the sample and fed into the trained meta-model to obtain the final output.

**Figure 27:**
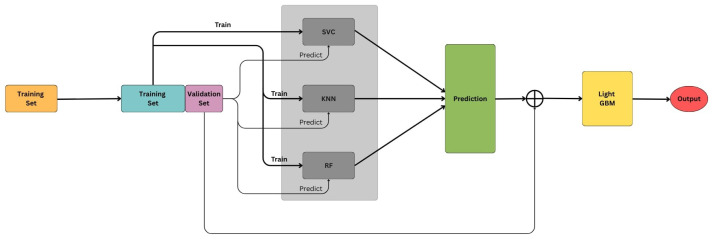
Model architecture.

This architecture, illustrated in Figure 27, allows the meta-model to leverage both the decision patterns of individual models and the raw feature information, improving generalization and robustness.

The hyperparameters for which the models performed best on the cross-validation sets are shown in Table 4.

### 3.4. Regression Models

For the regression task on age, we trained several widely known regressor models: Linear Regression [41], k-Nearest Neighbors (kNN) Regressor, Support Vector Regressor (SVR) [42], Random Forest (RF) Regressor, Xtreme Gradient Boosting (XGBoost) Regressor, and Light Gradient Boosting Machine (LightGBM) Regressor. The hyperparameters with the best performance on the cross-validation sets are shown in Table 5.

After training our models, we evaluated our classifier and regressor models on the test set for prediction of males and females and prediction of age, respectively. The results are shown in the following two subsections.

### 3.5. Performance Evaluation of Classifiers

**Figure 28:**
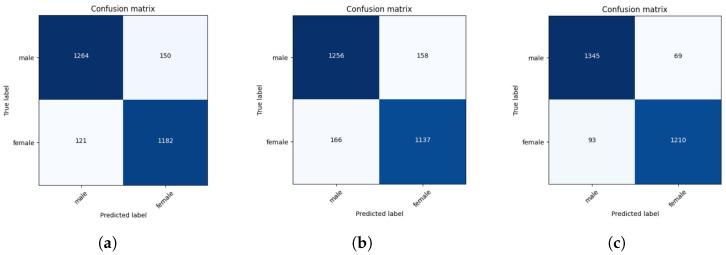
Confusion matrix of (**a**) Knn, (**b**) SVC, and (**c**) Stacking Ensemble.

**Figure 29:**
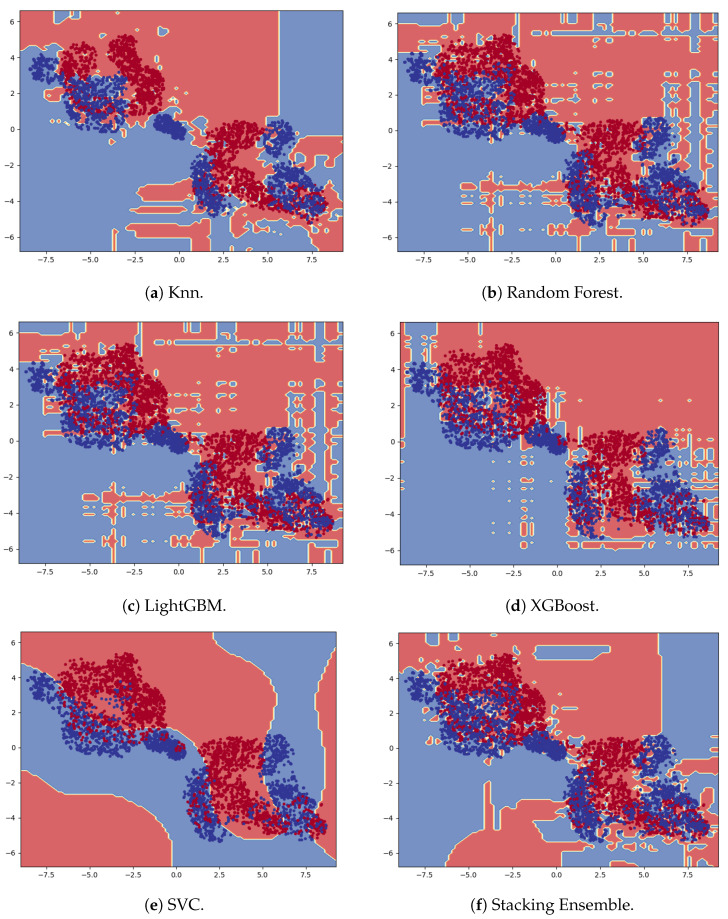
Decision boundary plots of classifier models (blue dots: female samples; red dots: male samples).

This section evaluates the performance of the trained models for sex classification (male and female) on the test set. As the dataset is balanced for male and female participants, we have used the following performance metrics used for classification: accuracy, F-1 Score, and AUC-ROC. AUC represents the area under the ROC curve, which plots the True Positive Rate (TPR) against the False Positive Rate (FPR). Table 6 shows the performance metrics on the test set for each model. Figure 28 shows the confusion matrix for the best three models. We plotted the decision boundary of each classifier shown in Figure 29. We have used t-Distributed Stochastic Neighbor Embedding (t-SNE) [43] with a dimensionality reduction technique to reduce the 30D feature space to a 2D feature space, as the the high-dimensional feature space is challenging to visualize.

Based on Table 6, Stacking Ensemble clearly outperforms the other models with 94% accuracy. K-Nearest Neighbor Classifier comes second, with 90% accuracy.

### 3.6. Performance Evaluation of Regressors

This section evaluates the performance of the trained models for regression task of age on the test set. The performance metrics used for classification are Mean Absolute Error (MAE), Mean Squared Error (MSE), and *R*^2^ score. The *R*^2^ score, also known as the coefficient of determination, is a statistical measure that indicates how well a regression model’s predictions approximate the actual data. It assesses the proportion of variance in the target variable that is predictable from the independent variables. The equations for the above metrics are shown below:

$$\mathrm{MAE}=\frac{1}{n}\sum_{i=1}^{n}\left|y_{i}-{\hat{y}}_{i}\right|,\;\mathrm{MSE}=\frac{1}{n}\sum_{i=1}^{n}{\left(y_{i}-{\hat{y}}_{i}\right)}^{2}$$

$$R^{2}=1-\frac{{\mathrm{SS}}_{\mathrm{res}}}{{\mathrm{SS}}_{\mathrm{tot}}},\;\mathrm{where}\;{\mathrm{SS}}_{\mathrm{res}}=\sum_{i=1}^{n}{\left(y_{i}-{\hat{y}}_{i}\right)}^{2},\;{\mathrm{SS}}_{\mathrm{tot}}=\sum_{i=1}^{n}{\left(y_{i}-\bar{y}\right)}^{2}$$

where,

- ${\mathrm{SS}}_{\mathrm{res}}$: Residual Sum of Squares—the sum of squared differences between observed values $y_{i}$ and predicted values ${\hat{y}}_{i}$.
- ${\mathrm{SS}}_{\mathrm{tot}}$: Total Sum of Squares—the sum of squared differences between observed values $y_{i}$ and their mean $\bar{y}$.

Table 7 shows the performance metrics on the test set for each model. We can see that K-Nearest Neighbor clearly outperforms the other models with 0.83 $R^{2}$ score. SVR comes second with $R^{2}$ score of 0.73.

### 3.7. Identifying the Best-Performing Age Group

**Figure 30:**
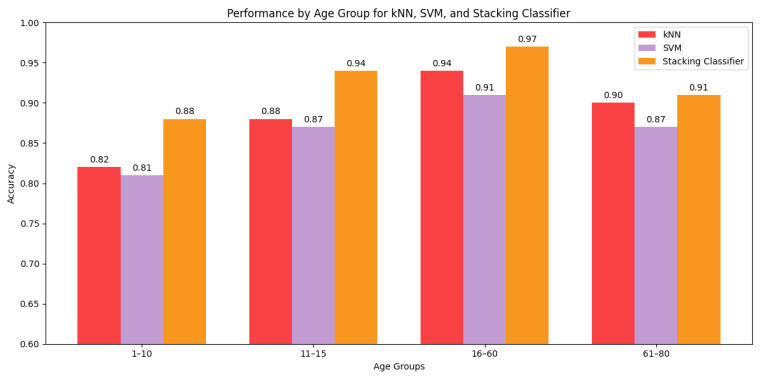
Comparison of accuracy across age groups of kNN, SVC, and Stacking Ensemble Classifier.

We have compared performance of best three classifier models in predicting sex across different age groups in Figure 30. Clearly, the models perform best in distinguishing males and females of age 11 to 60 because of their matured and consistent walking style. On the other hand, the performance dropped significantly for the group aged 1–10 years, as the walking patterns of male and female children of that age are usually similar and very inconsistent. For the group aged 61–80 years, walking patterns tend to become less stable due to various physical changes and health conditions, leading to a decline in model performance. However, the performance drop is not as significant as it is for the group aged 1–10 years.

**Figure 31:**
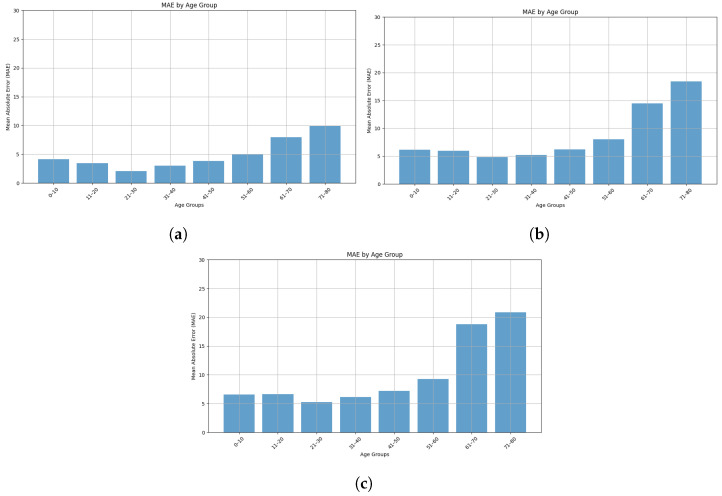
MAE by age group for (**a**) Knn, (**b**) SVR, and (**c**) LightGBM Regressor model.

We have also compared the performances of the best three regressor models in predicting age across different age groups in Figure 31. We see from the figure that groups aged 0–10 years through 51–60 years have relatively low MAE values with the lowest MAE for the 21–30 age group. However, the MAE is much higher for the group aged 61–80 years compared to the other age groups. As depicted in Figure 26, the dataset has much fewer samples from the 60–80 age group, so this imbalance could cause the model to perform poorly for the older age group. Moreover, physical changes and health conditions vary widely in people in the 60–80 age range, which may influence their walking patterns differently. So, it is difficult for the model to predict age without health-related information.

### 3.8. Finding the Optimal Position of the Sensor on the Body

As discussed in Section 2.2, the dataset contains samples acquired from three different sensor positions: center of the back and the right and left sides of the waist. We have compared the best three models’ performances for each sensor position for both sex and age prediction. The dataset contains a disproportionate number of samples from various sensor positions. Therefore, we have down-sampled to create datasets of the same size for training the models.

**Figure 32:**
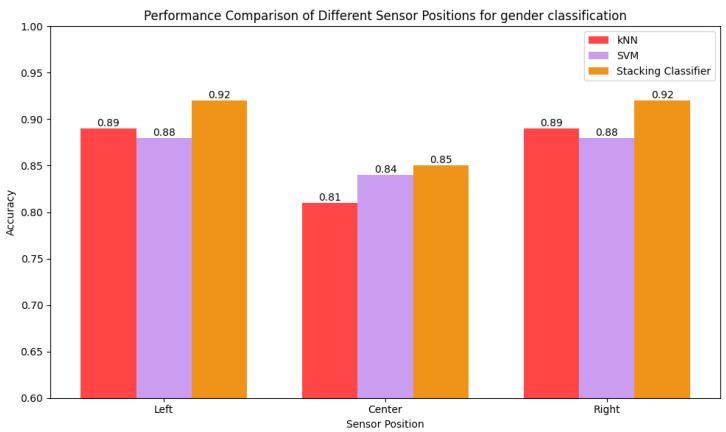
Comparison of accuracy of kNN, SVC, and stacking ensemble for different sensor positions.

**Figure 33:**
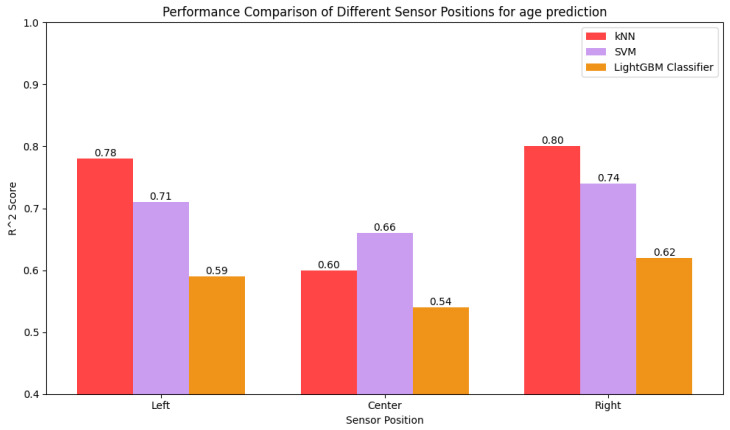
Comparison of $R^{2}$ score of kNN, SVR, and LightGBM Regressor for different sensor positions.

The performances of the models in predicting sex and age for different sensor positions are compared in Figure 32 and Figure 33, respectively. The center-positioned samples yield the lowest performance because the sensor fails to capture the dynamic range of the gait pattern due to being further from the motion of the leg while walking. In contrast, the models perform best on data collected from sensors positioned on both sides as the sides of the body experience more direct forces as the legs move. In cases of sex prediction, the performance is same for both the sides, but the right side outperforms the left side. The right leg is the most dominant leg for most people, so a sensor positioned on the right side is able to successfully capture different patterns for different ages.

## 4. Discussion

The primary objective of this study was to develop an effective and practical methodology for estimating a person’s sex and age from walking patterns using wearable sensors. We aimed to answer the following research questions:

1. Can meaningful features that reflect sex and age differences be extracted from inertial gait data?
2. Can traditional machine learning models accurately predict sex and estimate age based on those features?
3. Which sensor placements and demographic segments yield the most reliable results?

To address these questions, we analyzed gait signals captured from a large population (744 participants) using accelerometer and gyroscope sensors placed at the waist. We extracted a diverse set of statistics-, frequency-, jerk-, and correlation-based features and reduced them to the 30 most informative ones using Recursive Feature Elimination (RFE). These features were then used to train various classification and regression models.

Our findings demonstrate that traditional ML models, when trained on a carefully engineered feature set, can outperform existing solutions in both sex classification and age estimation. The contrast between our work and that of recent studies is presented in Table 8, which highlights key differences in dataset size, feature design, methodology, and performance. As shown in the table, our approach achieved a sex prediction accuracy of 94.4% and an age estimation *R*^2^ score of 0.83, outperforming several previous works. Notably, although some previous studies [16,17,19] reported comparable sex classification accuracies, their work was based on relatively small datasets compared to ours, which includes 744 participants. In particular, Sabir et al. [17] employed a deep learning model (RNN-LSTM) on a dataset of only 60 subjects. In contrast, our approach leverages a substantially larger dataset and employs interpretable, handcrafted features in conjunction with traditional machine learning algorithms, offering greater transparency and practicality for real-world applications.

(a) **Feature Relevance and Interpretability:** While Ahad et al. [25] reported strong performance using deep learning models like temporal convolutional networks and Angle-Embedded Gait Dynamic Images (AE-GDI), they emphasized that such models act as “black boxes” and offer limited interpretability. In contrast, our use of handcrafted features allows clear physiological interpretations that are useful for clinical or real-world deployment.
(b) **Model Performance and Generalization Behavior:** Our findings demonstrate that traditional machine learning models, when trained on a carefully engineered feature set, can outperform many existing deep-learning-based solutions in both sex classification and age estimation. As highlighted in Table 8, our model achieved a sex prediction accuracy of 94.4% and an age estimation $R^{2}$ score of 0.83. This surpasses the results reported in previous studies [16,17,19], many of which relied on significantly smaller datasets or less interpretable deep models.
  For sex classification, the Stacking Ensemble model achieved the highest performance, followed by kNN and SVC. Interestingly, the decision boundary of SVC was the smoothest, suggesting better generalization with lower overfitting risk, whereas the more complex boundary formed by the Stacking Ensemble model likely contributed to its superior accuracy by capturing intricate decision regions—though this came at the potential cost of increased sensitivity to outliers or distribution shifts.
  In age estimation, the kNN Regressor yielded the best $R^{2}$ score, followed by SVR and LightGBM. However, we observed a notable performance drop in all models for the elderly age group (61–80 years). This decline is primarily attributed to the class imbalance in the dataset—a known challenge in regression with continuous targets. Our current approach does not yet incorporate reweighting or SMOTER, but we acknowledge that addressing this imbalance is critical for achieving more consistent performance across all age groups.
(c) **Model Robustness Across Age Groups:** From our research, we have found that male and female children aged 1–10 years have very similar walking patterns. Therefore, they form the most challenging age group for accurate sex prediction, with the highest obtained accuracy being 88%. As they grow into adults, their walking patterns diverge significantly due to physiological differences between males and females. For this reason, the adult age group (16–60) is the easiest for accurate sex prediction with highest accuracy of 97%. However, as individuals enter old age (61–80), the stability of their walking pattern often deteriorates due to physical conditions and health issues. Nevertheless, the differences are significant enough for yielding a modest performance of 91%.
  In case of age estimation, children (1–10 years) and adults (11–60 years) are the easiest to predict accurately due to their distinct walking patterns. Children’s walking patterns are characterized by short, inconsistent strides and frequent erratic motions, whereas the walking patterns of adults demonstrate more stable and consistent strides. However, the performance significantly declines for the older age group (61–80) because of the wide variations in their walking patterns due to wide-ranging health conditions. Moreover, the imbalance in the dataset, with very few samples representing this older age group, also contributes to the reduced performance. This finding aligns with those of Van Hamme et al. [26], who noted that model accuracy in older populations tends to degrade unless specific balancing strategies (e.g., stratified sampling or segment-wise averaging) are applied.
(d) **Optimal Sensor Positioning:** Our analysis reveals that the left and right sides of the waist just above the legs are the optimal sensor placements for predicting sex and age. Sensors positioned at these locations are able to collect more direct forces and vibrations during the leg movements of walking and capture finer details of the movements of both the legs and the waist. In contrast, sensors placed at the center of the back, being farther from the primary sources of motion, fail to capture distinct patterns related to leg movements, resulting in poor performance. This conclusion is consistent with those of both Ahad et al. [25] and Van Hamme et al. [26], who found that hip or lower trunk placements capture richer gait signals than chest or ankle sensors.

## 5. Limitations

While this study demonstrates strong performance in sex classification and age estimation using wearable sensors, several limitations should be acknowledged:

1. **Dataset Diversity:** The dataset used (the OU-ISIR Inertial Gait Database) consists of participants from a single ethnicity and geographic region. As a result, the model’s generalizability to populations with different cultural or physical traits is uncertain.
2. **Sensor Position Constraints:** Although we compared performance across three sensor placements (center, right, and left waist), the study focused solely on the waist area. Gait dynamics may differ significantly when sensors are placed on the foot, ankle, or wrist, which were not explored here.
3. **Age Group Imbalance:** The dataset is imbalanced in terms of age distribution; in particular, we had fewer samples from older adults (ages 61–80). This imbalance affects the accuracy of the conclusions that can be drawn for elderly populations.
4. **Limited Contextual Information:** The model does not account for contextual or physical variables such as height, weight, health conditions, or footwear, which can significantly affect gait. Incorporating these factors could improve performance, especially in sex and age estimation.
5. **Activity Scope:** The analysis is restricted to level-ground walking. Real-world walking patterns may vary significantly in different conditions, such as stair climbing, uneven terrain, or running, which were not included in this study.

## 6. Conclusions

This study set out to develop a robust and interpretable method for sex classification and age estimation using gait data collected from wearable inertial sensors. Through a comprehensive approach that combined carefully engineered features with traditional machine learning models, we were able to address the limitations of previous works and achieve state-of-the-art results on the OU-ISIR inertial gait dataset.

To summarize, our approach achieved 94.4% accuracy in sex prediction and an *R*^2^ score of 0.83 in age estimation, outperforming previous studies in both performance and generalizability. We demonstrated that meaningful physical and statistical characteristics—such as step interval, acceleration variability, and frequency domain features—can be extracted from time–series sensor data and used to build accurate predictive models. Our analysis also highlighted how sex and age influence gait in distinct ways and how model performance varies across age groups and sensor placements.

This work addresses a clear gap in the existing literature: while many prior studies either relied on small datasets or deep learning models, our study shows that interpretable feature engineering, coupled with traditional ML, can yield competitive results on a large and diverse dataset. By analyzing the specific features that drive model performance, we offer a clearer understanding of the underlying biomechanical patterns that differentiate individuals by sex and age.

The implications of our findings are significant. Beyond making academic contributions, the proposed framework could be applied in a variety of real-world domains—such as smart surveillance systems, health monitoring, personalized user interfaces, and access control based on demographic profiling. Importantly, the use of wearable sensors like those in smartphones or fitness devices makes this solution highly practical and accessible.

Looking ahead, this research opens up new avenues for gait-based soft biometrics. Future studies could expand this work by incorporating additional physical or medical attributes such as height, weight, or health conditions to improve age estimation in elderly individuals. Further, exploring multi-modal sensing, sensor fusion, or testing the models across more diverse populations and environments could broaden the applicability and robustness of such systems.

## Figures and Tables

**Table 1:** Selected features for sex and age prediction (common features between both sets are marked with ^†^).

Category	Sex Prediction Feature Set	Age Prediction Feature Set
Time	accl-net-positive-peak-interval-mean ^†^	accl-net-positive-peak-interval-mean ^†^
	accl-net-positive-peak-interval-std	accl-x-mean
	accl-x-skewness	accl-y-mad
	accl-y-kurtosis	accl-z-mad
	accl-y-res-std	gyro-x-iqr
	accl-y-positive-peak-interval-mean	gyro-y-negative-peak-val-mean ^†^
	accl-y-peak-interval-std	gyro-z-kurtosis
	accl-z-negative-peak-val-mean	
	accl-z-negative-peak-interval-std	
	gyro-x-positive-peak-val-mean	
	gyro-y-positive-peak-interval-mean	
	gyro-y-negative-peak-val-mean ^†^	
	gyro-z-positive-peak-val-mean	
	gyro-z-negative-peak-interval-std	
Frequency	accl-net-skew-freq-psd	accl-net-percentage-power-delta-band-psd
	accl-x-percentage-power-alpha-band-psd ^†^	accl-x-percentage-power-alpha-band-psd ^†^
	accl-x-skewness-freq ^†^	accl-x-percentage-power-beta-band-psd
	accl-y-percentage-power-beta-band-psd	accl-x-skewness-freq ^†^
	accl-z-percentage-power-delta-band-psd	accl-y-percentage-power-alpha-band-psd
	accl-z-percentage-power-beta-band-psd	accl-z-kurt-freq-psd
	gyro-y-bandwidth-psd-lower-bound	accl-z-percentage-power-theta-band-psd
	gyro-z-percentage-power-alpha-band-psd	gyro-y-percentage-power-theta-band-psd
		gyro-y-percentage-power-gamma-band-psd
		gyro-z-percentage-power-theta-band-psd
Jerk	-	gyro-z-jerk-iqr, gyro-z-jerk-skewness
Correlation	accl-x-accl-y-spearman ^†^	accl-x-accl-y-spearman ^†^
	accl-x-accl-z-spearman ^†^	accl-x-accl-z-spearman ^†^
	accl-x-gyro-z-spearman ^†^	accl-x-gyro-x-spearman
	accl-y-accl-z-spearman	accl-x-gyro-y-spearman
	accl-y-gyro-z-spearman ^†^	accl-x-gyro-z-spearman ^†^
	accl-net-gyro-x-spearman	accl-y-gyro-x-spearman
	gyro-x-gyro-y-spearman ^†^	accl-y-gyro-z-spearman ^†^
	gyro-x-gyro-z-spearman	accl-z-accl-net-spearman
		accl-z-gyro-x-spearman
		gyro-x-gyro-y-spearman ^†^
		gyro-y-gyro-z-spearman

**Table 2:** Feature names for sex classification and their corresponding analytical names (Forward Tilt, Lateral Twist, and Vertical Swing denote rotational motion around the x, y, and z axes, respectively.)

No.	Feature Name	Analytical Name
1	accl-net-positive-peak-interval-mean	Avg Step Interval
2	accl-net-positive-peak-interval-std	Step Interval Variability
3	accl-x-skewness	Skewness of Lateral Acceleration
4	accl-y-kurtosis	Kurtosis of Vertical Acceleration
5	accl-y-res-std	Deviation of Vertical Acceleration
6	accl-y-positive-peak-interval-mean	Avg Interval Between Peaks in Vertical Acceleration
7	accl-y-peak-interval-std	Variability in Interval Between Peaks of Vertical Acceleration
8	accl-z-negative-peak-val-mean	Lower Bound of Forward Acceleration
9	accl-z-negative-peak-interval-std	Variability in Interval Between Peaks in Forward Deceleration
10	gyro-x-positive-peak-val-mean	Upper Bound of Forward Tilt Motion
11	gyro-y-positive-peak-interval-mean	Avg Interval Between Peaks of Lateral Twist Motion
12	gyro-y-negative-peak-val-mean	Lower Bound of Lateral Twist Motion
13	gyro-z-positive-peak-val-mean	Upper Bound of Vertical Swing Motion
14	gyro-z-negative-peak-interval-std	Variability in Interval Between Peaks of Vertical Swing Motion
15	accl-net-skew-freq-psd	Skewness of frequency of Net Acceleration
16	accl-x-percentage-power-alpha-band-psd	Power Concentration of Lateral Acceleration in Alpha Band
17	accl-x-skewness-freq	Skewness of frequency of Lateral Motion
18	accl-y-percentage-power-beta-band-psd	Power Concentration of Vertical Acceleration in Beta Band
19	accl-z-percentage-power-delta-band-psd	Power Concentration of Forward Acceleration in Delta Band
20	accl-z-percentage-power-beta-band-psd	Power Concentration of Forward Acceleration in Beta Band
21	gyro-y-bandwidth-psd-lower-bound	Lower Bound of Bandwidth of Lateral Twisting Motion
22	gyro-z-percentage-power-alpha-band-psd	Power Concentration of Swing Motion in Alpha Band
23–30	Multiple Correlation Features	Motion Correlation Metrics—capturing relationships between acceleration and rotation across the forward, lateral, and vertical axes

**Table 3:** Feature names for age prediction and their corresponding analytical names (Forward Tilt, Lateral Twist, and Vertical Swing denote rotational motion around the x, y, and z axes, respectively).

No.	Feature Name	Analytical Name
1	accl-net-positive-peak-interval-mean	Avg Step Interval
2	accl-x-mean	Avg Forward Acceleration
3	accl-y-mad	Deviation of Vertical Acceleration
4	accl-z-mad	Deviation of Forward Acceleration
5	gyro-x-iqr	Range of Forward Tilt Motion
6	gyro-y-negative-peak-val-mean	Lower Bound of Lateral Twist Motion
7	gyro-z-kurtosis	Kurtosis of Vertical Swing Motion
8	accl-net-percentage-power-delta-band-psd	Power Concentration of Net Acceleration in Delta Band
9	accl-x-percentage-power-alpha-band-psd	Power Concentration of Lateral Acceleration in Alpha Band
10	accl-x-percentage-power-beta-band-psd	Power Concentration of Lateral Acceleration in Beta Band
11	accl-x-skewness-freq	Skewness of frequency of Lateral Acceleration
12	accl-y-percentage-power-alpha-band-psd	Power Concentration of Vertical Acceleration in Alpha Band
13	accl-z-kurt-freq-psd	Kurtosis of frequency of Forward Acceleration
14	accl-z-percentage-power-theta-band-psd	Power Concentration of Forward Acceleration in Theta Band
15	gyro-y-percentage-power-theta-band-psd	Power Concentration of Lateral Twist Motion in Theta Band
16	gyro-y-percentage-power-gamma-band-psd	Power Concentration of Lateral Twist Motion in Gamma Band
17	gyro-z-percentage-power-theta-band-psd	Power Concentration of Vertical Swing Motion in Theta Band
18	gyro-z-jerk-iqr	Range of rate of change of Vertical Swing Motion
19	gyro-z-jerk-skewness	Skewness of rate of change of Vertical Swing Motion
20–30	Multiple Correlation Features	Motion Correlation Metrics—capturing relationships between acceleration and rotation across the forward, lateral, and vertical axes

**Table 4:** Hyperparameters of classifier models.

Model	Hyperparameters
LR	C = 0.1, solver = ‘lbfgs’
SVC	C = 10, kernel = ‘rbf’
kNN	n_neighbors = 3, weights = ‘distance’, metric = ‘manhattan’
RF	n_estimators = 100, bootstrap = False, criterion = ‘gini’
XGBoost	n_estimators = 500, learning_rate = 0.1, subsample = 0.7, max_depth = 10
LightGBM	n_estimators = 500, learning_rate = 0.1, subsample = 0.4
Stacking Ensemble	passthrough=True
Base Models	SVC, kNN, RF (same as above)
Meta Model (LightGBM)	n_estimators = 100, learning_rate = 0.1, subsample = 0.4

**Table 5:** Hyperparameters of regressor models.

Model	Hyperparameters
SVR	C = 150, kernel = ‘rbf’, epsilon = 0.001
kNN	n_neighbors = 3, metric = ‘manhattan’, weights = ‘distance’
RF	n_estimators = 100
XGBoost Regressor	n_estimators = 300, learning_rate = 0.1, max_depth = 10, subsample = 0.8
LightGBM Regressor	n_estimators = 500, learning_rate = 0.1, max_depth = −1, subsample = 0.5

**Table 6:** Performance evaluation of classifiers.

Classifier	Accuracy	AUC-ROC
LR	0.75	0.82
SVC	0.88	0.95
kNN	0.90	0.97
RF	0.83	0.91
XGBoost	0.87	0.94
LightGBM	0.87	0.94
**Stacking Ensemble**	**0.94**	**0.98**

**Table 7:** Performance evaluation of regression models.

Regressor Model	MAE	RMSE	*R*^2^ Score
Linear Regression	11.15	198	0.34
**kNN**	**3.53**	**51.1**	**0.83**
SVR	6.38	82	0.73
RF	8.74	137.8	0.54
XGBoost	7.57	115.5	0.61
LightGBM	7.23	104.3	0.65

**Table 8:** Comparison of related works.

Author(s)	Participants	Features	Method/Model	Accuracy
Jain et al. [16]	109 subjects	Histogram of Oriented Gradients (HOG)	Bootstrap Aggregating Classifier	- 94.44% (fast walking) - 91.07% (normal walking)
Sabir et al. [17]	30 males, 30 females	38 extracted features	RNN-LSTM	94.11%
Davarci et al. [18]	100 participants	Features analyzing accelerometer correlations	Hybrid model	- 85% (sitting/standing) - 83% (walking)
Meena et al. [19]	109 subjects	Reduced triaxial acceleration (PCA-based)	Bagging Trees	96.3%
Khabir et al. [22]	Not specified	88 extracted features	SVM (sex) Decision Tree (age)	- 84.76% (sex classification) - $R^{2}$ = 0.64 (age estimation)
Pathan et al. [23]	1556 subjects	84 features (using NCA)	Bagging Trees	87.85% (sex prediction)
This study	744 participants	30 features	Sex—stacking ensemble Age—KNN	94.4% (sex prediction) - $R^{2}$ = 0.83 (age estimation)

## Data Availability

Restrictions apply to the availability of these data. Data were obtained from the Institute of Scientific and Industrial Research (ISIR), Osaka University (OU), and are available at http://www.am.sanken.osaka-u.ac.jp/BiometricDB/InertialGait.html (accessed on 26 May 2025) with the permission of the Institute of Scientific and Industrial Research (ISIR), Osaka University (OU).

## Abbreviations

The following abbreviations are used in this manuscript:

AI	artificial intelligence
OU-ISIR	Institute of Scientific and Industrial Research (ISIR), Osaka University (OU)
RNN	Recurrent Neural Network
LSTM	Long Short-term Memory
PCA	Principal Component Analysis
SVM	Support Vector Machine
NCA	Neighborhood Component Analysis
MEMS	Micro-Electromechanical System
IMU	Inertial Measurement Unit
FFT	Fast Fourier Transform
PSD	power spectral density
std	standard deviation
mad	mean absolute deviation
iqr	inter-quartile range
accl	accelerometer
gyro	gyroscope
RFE	Recursive Feature Elimination
SVR	Support Vector Regression
